# Interpretable machine learning model integrating contrast-enhanced CT environmental radiomics and clinicopathological features for predicting postoperative recurrence in lung adenocarcinoma: a retrospective pilot study

**DOI:** 10.3389/fonc.2025.1601674

**Published:** 2025-05-23

**Authors:** Song Lin, Yanli Niu, Lina Song, Yingjian Ye, Jinfang Yang, Junjie Liu, Xin Zhou, Peng An

**Affiliations:** ^1^ Department of Radiology and Surgery, Xiangyang No.1 People’s Hospital, Hubei University of Medicine, Xiangyang, China; ^2^ Department of Medical Cosmetology, Anesthesiology, Oncology, and Epidemiology, Xiangyang Key Laboratory of Maternal-fetal Medicine on Fetal Congenital Heart Disease, Xiangyang No. 1 People’s Hospital, Hubei University of Medicine, Hubei, People's Republic of China (P.R.C), Xiangyang, Hubei, China

**Keywords:** lung adenocarcinoma, contrast-enhanced CT, radiomics, machine learning, recurrence prediction

## Abstract

**Purpose:**

This study aims to develop an interpretable predictive model combining contrast-enhanced CT (CECT) radiomics features with clinicopathological parameters to assess 3-year recurrence risk after surgery for lung adenocarcinoma (LA).

**Methods:**

A retrospective cohort of 350 LA patients (126 recurrence, 224 non-recurrence) from Xiangyang NO.1 People’s Hospital (2016–2023) was included. Radiomics features were extracted from arterial and venous phase CECT images using 3D Slicer’s Radiomics plugin. Features with intraclass correlation coefficient (ICC > 0.75) were selected, followed by LASSO regression with cross-validation to generate radiomics scores (Radscore3 for intratumoral and Radscore4 for peritumoral regions). Clinical variables (sex, heterogeneous enhancement, pleural invasion, Ki67) were integrated via chi-square/t-test analysis. Ten machine learning algorithms (e.g., XGBoost, CatBoost, Random Forest) were trained on a stratified 7:3 split (training: n=245; testing: n=105) with five-fold cross-validation. Model performance was evaluated using ROC curves (AUC), calibration curves, decision curve analysis (DCA), and a nomogram.

**Results:**

Univariate analysis identified sex (OR=1.66, p=0.02), heterogeneous enhancement (OR=4.32, p<0.05), visceral pleural invasion (OR=4.75, p<0.05), Radscore3 (OR=356.17, p<0.05), Radscore4 (OR=1529.16, p<0.05), and Ki67 (OR=1.09, p=0.01) as significant predictors. Among machine learning models, CatBoost achieved superior performance (AUC=0.883, 95% CI:0.811–0.955) compared to logistic regression (AUC=0.877, 95% CI:0.804–0.949) in test set. Calibration curves demonstrated high consistency between predicted and observed recurrence risks, while DCA indicated clinical utility at threshold probabilities >0.17. SHAP analysis highlighted heterogeneous enhancement, visceral pleural invasion, Radscore3/4, and Ki67 as key contributors. The nomogram integrated these factors, enhancing model interpretability and clinical applicability.

**Conclusion:**

The CatBoost model integrating CECT environmental radiomics and clinicopathological parameters effectively predicts postoperative LA recurrence, supporting personalized adjuvant therapy decisions. Its interpretable framework emphasizes tumor heterogeneity (Radscore3/4) as a critical prognostic biomarker, providing mechanistic insights into LA recurrence.

## Introduction

Lung adenocarcinoma (LA), the predominant subtype of non-small cell lung cancer, remains a formidable clinical challenge due to its high postoperative recurrence rates and heterogeneous responses to adjuvant therapies. While the AJCC TNM staging system serves as the cornerstone for recurrence risk stratification, its inability to address molecular heterogeneity and deliver personalized prognostic insights has become increasingly apparent. Emerging evidence highlights the limitations of traditional biomarkers such as serum CEA, CA125, and CYFRA21-1, whose diagnostic accuracy is compromised by tumor biological complexity and insufficient specificity. Recent advancements in multimodal imaging and machine learning (ML) have opened new avenues for precision prognostication. For instance, deep learning models leveraging whole-slide pathological images have demonstrated enhanced recurrence prediction by quantifying tumor-infiltrating lymphocyte distributions and stromal ratios. However, these approaches rely on costly postoperative specimens, lack preoperative applicability, and suffer from interpretability deficits inherent to “black-box” architectures. Single-cell transcriptomic analyses further reveal immune-related mRNA signatures (e.g., 16-gene panels) and metabolic reprogramming pathways (e.g., 3S-MMR models optimized via genetic algorithms) as independent predictors of recurrence in stage I–III LA. Yet, these omics-driven strategies fail to integrate imaging biomarkers with molecular profiles, limiting their clinical utility (1–4). To bridge these gaps, we propose an interpretable radiomics-clinicopathological fusion framework with three pivotal innovations: 1. Multiphase Radiomics Profiling: By extracting intratumoral (Radscore3) and peritumoral (Radscore4) radiomics features from arterial/venous-phase contrast-enhanced CT, we quantify tumor heterogeneity and enhancement dynamics, overcoming the constraints of conventional 2D morphological metrics. 2. Explainable ML Architecture: Ten algorithms (including CatBoost and XGBoost) are systematically evaluated, with SHAP (SHapley Additive exPlanations) analysis elucidating critical predictors such as pleural invasion and Ki67 index, thereby addressing the transparency crisis in deep learning. 3. Multimodal Integration: A hybrid prediction network synergizing sex, Ki67 expression, tumor enhancement heterogeneity, and radiomics scores achieves superior performance. This study not only advances LA recurrence prediction but also provides mechanistic insights into tumor microenvironment heterogeneity, paving the way for precision oncology applications (5–7).

## Research methodology

### Data source and eligibility criteria

**Figure 1:**
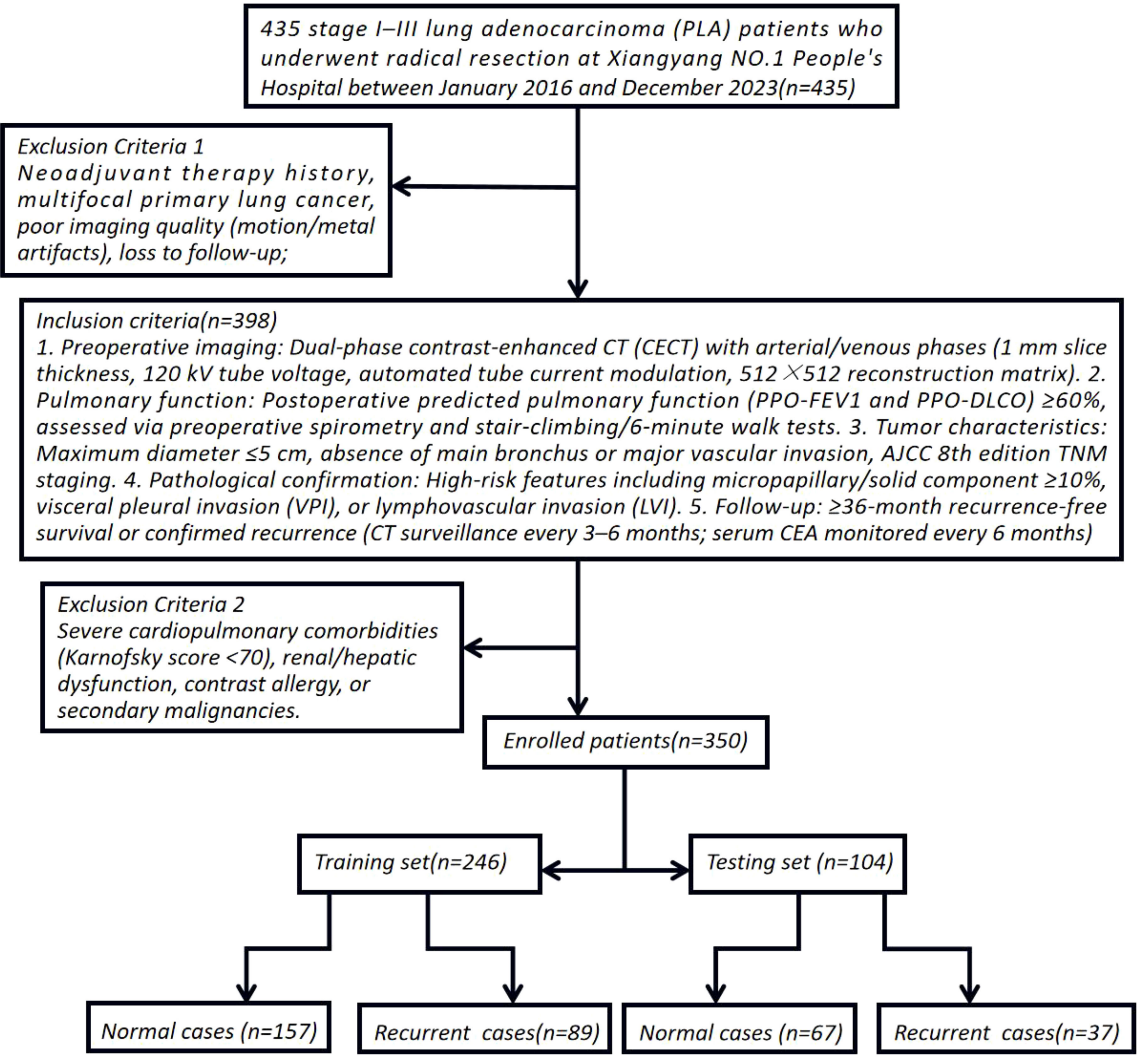
Schematic diagram of study enrollment criteria and cohort stratification.

This single-center retrospective cohort study enrolled 435 stage I–III lung adenocarcinoma (LA) patients who underwent radical resection at Xiangyang NO.1 People’s Hospital between January 2016 and December 2023. To simulate multicenter validation, the cohort spanned three geographically distinct hospital branches (20–30 km apart), introducing spatial and demographic heterogeneity akin to external validation cohorts. Inclusion Criteria: 1. Preoperative imaging: Dual-phase contrast-enhanced CT (CECT) with arterial/venous phases (1 mm slice thickness, 120 kV tube voltage, automated tube current modulation, 512×512 reconstruction matrix). 2. Pulmonary function: Postoperative predicted pulmonary function (PPO-FEV1 and PPO-DLCO) ≥60%, assessed via preoperative spirometry and stair-climbing/6-minute walk tests. 3. Tumor characteristics: Maximum diameter ≤5 cm, absence of main bronchus or major vascular invasion, AJCC 8th edition TNM staging. 4. Pathological confirmation: High-risk features including micropapillary/solid component ≥10%, visceral pleural invasion (VPI), or lymphovascular invasion (LVI). 5. Follow-up: ≥36-month recurrence-free survival or confirmed recurrence (CT surveillance every 3–6 months; serum CEA monitored every 6 months). Exclusion Criteria: Neoadjuvant therapy history, multifocal primary lung cancer, poor imaging quality (motion/metal artifacts), loss to follow-up, severe cardiopulmonary comorbidities (Karnofsky score <70), renal/hepatic dysfunction, contrast allergy, or secondary malignancies. Cohort Stratification: After exclusions, 350 enrolled patients were stratified by smoking history, hypertension/diabetes, gender, age, BMI, neutrophil-to-lymphocyte ratio (NLR), platelet-to-lymphocyte ratio (PLR), VPI, preoperative CEA (>5 ng/mL), and postoperative Ki67 index (immunohistochemical quantification). These patients were randomly allocated to training (n=245) and independent testing (n=105) sets at a 7:3 ratio to ensure balanced representation of risk factors (8–10) (**Figure 1**). Ethical Compliance: Approved by the Institutional Review Board of Xiangyang NO.1 People’s Hospital (Approval No.: XYYYE20240011). All participants provided informed consent, with anonymized data stored in encrypted databases compliant with GDPR standards.

### CECT radiomics feature extraction

**Figure 2:**
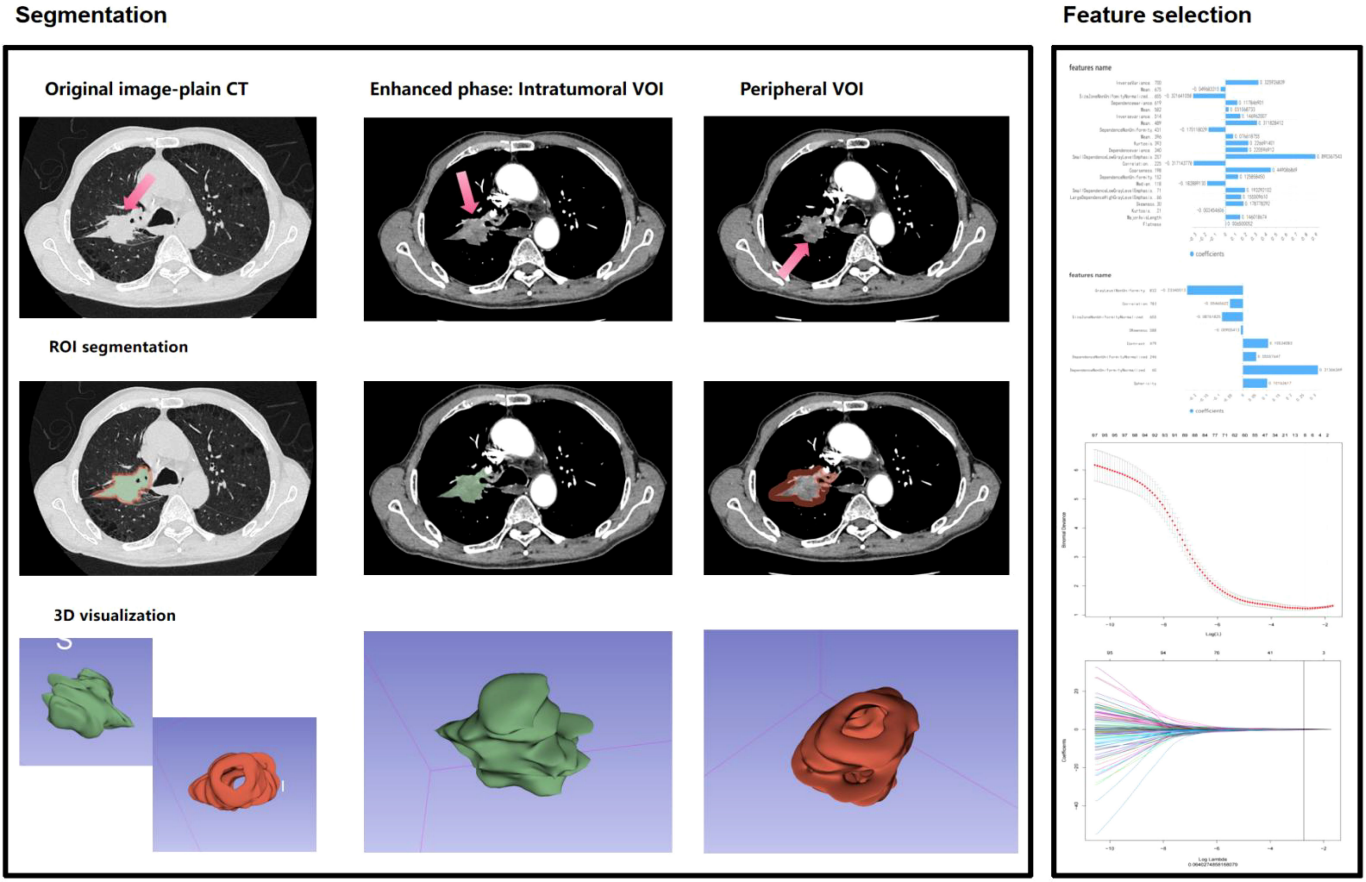
Workflow for environmental radiomics feature extraction from contrast-enhanced CT scans of lung adenocarcinoma (LA) lesions, integrating intratumoral heterogeneity (Radscore3) and peritumoral microenvironment dynamics (Radscore4).

Enhanced CT Lung Scanning Protocol for Toshiba Aquilion ONE 320-Detector CT based on device specifications and clinical guideline: 1. Pre-Scan Preparation: Fasting for 6–8 hours, remove metallic objects from the chest, obtain informed consent, establish IV access via the median cubital vein. 2. Positioning: Supine position, arms raised above the head. Scan baseline: 2–3 cm above the clavicle; coverage from thoracic inlet to 2–3 cm below the costophrenic angle. 3. Scan Parameters: Mode: Dynamic Volume CT (DVCT) with 0.5 mm slice thickness; 160 mm/rotation coverage. Full lung scan completed in 0.35 sec. Technical settings: 120 kV, 100 mA, matrix 512×512, pitch 1.0875.Reconstruction: Bone algorithm (lung tissue) combined with soft-tissue algorithm (mediastinum). 4. Contrast Protocol: Dosage: 1.0-1.2 ml/kg non-ionic iodinated contrast agent; injection rate 2.5-3.5 ml/s. Timing: Arterial phase: 25–35 sec (empirical method) or 6 sec after threshold triggering (bolus tracking), Venous phase: 50–70 sec. 5. Data Archiving: All images saved in DICOM format to a portable hard drive. This protocol leverages the Toshiba 320-detector CT’s volumetric scanning capability for full-lung coverage in a single rotation, ensuring diagnostic accuracy with minimized motion artifacts and radiation exposure. ComBat harmonization was applied to mitigate inter-scanner variability. Combat harmonization (via `neuroCombat` R package v3.0) was applied to radiomic features to correct inter-scanner variability, while Z-score normalization standardized feature scales post-harmonization. Image Acquisition and Preprocessing: 1. Dual-phase registration: Arterial and venous phase images were spatially aligned using the Elastix toolkit’s elastic registration algorithm to ensure spatiotemporal consistency of tumor regions.2. Standardization: Z-score normalization combined with ComBat batch-effect correction to mitigate scanner and window-setting variations. Feature Extraction: A total of 1,718 radiomics features were extracted via 3D Slicer’s PyRadiomics module, including: Morphological: Sphericity, surface-to-volume ratio, 3D fractal dimension;- Second-order textures: Gray-level co-occurrence matrix (GLCM) contrast/entropy, gray-level run-length matrix (GLRLM) short-run emphasis;- Dynamic enhancement: ΔHU (arterial-to-venous CT value change rate), Gabor-filtered energy spectrum heterogeneity. Feature Selection: LASSO regression with recursive feature elimination (RFE) and 10-fold cross-validation identified robust predictors, λ selected via 10-fold cross-validation minimizing mean squared error. Two radiomics signatures were constructed using radiomics score(Radscore): Radscore3 (intratumoral): Arterial-phase minus venous-phase features quantifying internal heterogeneity. Radscore4 (peritumoral): Peri-tumor enhancement dynamics reflecting microenvironmental alterations (**Figure 2**) (11–13).

**Key Technical Innovations:** 1. Multiphase Radiomics: Dual-phase CECT captures tumor vascular heterogeneity, overcoming limitations of static 2D morphological metrics. 2. Batch-Effect Mitigation: ComBat harmonization ensures cross-scanner reproducibility, aligning with IBSI radiomics standards. 3. Dynamic Signatures: ΔHU and Gabor filters enhance sensitivity to angiogenesis patterns, validated in recent studies on LUAD recurrence. This methodology integrates rigorous clinical stratification with advanced radiomics, providing a reproducible framework for recurrence prediction in resectable LA.

### Statistical methodology and model construction

1. Normality Assessment: Continuous variables were evaluated for normality using the Kolmogorov-Smirnov (K-S) test. This method partitions data into equiprobability intervals to compare observed versus theoretical frequencies, with the cumulative distribution difference quantified via the K-S statistic (α=0.05). Variables failing normality assumptions were analyzed using nonparametric alternatives.2. Group Comparisons:- Independent t-tests: Applied for normally distributed variables to compare means between recurrence and non-recurrence groups, preceded by Levene’s test for homogeneity of variance.- Mann-Whitney U test: Used for non-normally distributed variables to assess group differences in medians.3. Logistic Regression with Feature Selection: A stepwise regression framework (bidirectional elimination) identified predictors of recurrence using maximum likelihood estimation. Odds ratios (ORs) with 95% confidence intervals were computed, prioritizing variables with Akaike Information Criterion (AIC) optimization.4. Machine Learning model construction: Algorithm Comparison: Ten models (CatBoost, XGBoost, LightGBM, SVM, KNN, GBM, Random Forest, Neural Networks, etc.) were trained using stratified 5-fold cross-validation.- Optimization Metrics: Area under the ROC curve (AUC) prioritized for class imbalance robustness, supplemented by Brier score for calibration accuracy.- Interpretability: SHAP (SHapley Additive exPlanations) values quantified feature contributions, with attention mechanisms dynamically weighting multimodal features.5. Model Performance Evaluation: Calibration Curves: Assessed via Hosmer-Lemeshow goodness-of-fit test, with Brier score quantifying deviations between predicted probabilities and observed outcomes. Decision Curve Analysis (DCA): Net benefit curves evaluated clinical utility across threshold probabilities, highlighting ranges where model-guided decisions outperform treat-all or treat-none strategies.6. Prognostic Nomogram Development: A multivariable scoring system integrated SHAP-derived clinical coefficients and radiomics weights. Bootstrap resampling (1,000 iterations) validated the C-index stability, while external cohort testing confirmed generalizability. Developing Nomograms in R: 1. Install the `survival` and `rms` packages. 2. Steps: Load the dataset and build a logistic regression model. Generate a nomogram object using the `nomogram()` function. Plot the nomogram with `plot()`, displaying variable contributions and predicted probabilities via scale lines (14, 15).

## Results

### Patient characteristics ​​

The study included 224 non-recurrent and 126 recurrent patients. Baseline features (age: 37–72 years, BMI: 17.3-32.0, smoking history) showed no significant differences (P>0.05). However, the recurrent group had fewer males (59 vs. 133 cases), higher tumor enhancement heterogeneity (predominant outflow pattern), pleural invasion (61 vs. 52 cases), and RadScore 3/4 prevalence (all P<0.05).

### Radiomics feature selection and model development

From 1,718 radiomics features extracted from contrast-enhanced CT, 1,686 features with intraclass correlation coefficients (ICC) >0.75 were retained, with the median and range of ICC presenting 0.90, 0.86~0.99. LASSO regression (λ=0.064027) combined with recursive feature elimination (RFE) and cross-validation identified two core signatures: - Radscore3 (intratumoral heterogeneity): Quantified venous-phase HU change rate (ΔHU=32.5 ± 4.7) and morphological parameters (OR=356.17), reflecting tumor internal heterogeneity.- Radscore4 (peritumoral heterogeneity): Derived from Gray-Level Co-Occurrence Matrix (GLCM) and Gray-Level Size Zone Matrix (GLSZM) features, capturing spiculation (SizeZoneNonUniformityNormalized…655, weight=0.321; SmallDependenceLowGrayLevelEmphasis.257, weight=0.890) and peritumoral vascular convergence (Coarseness.198, weight=0.449; Correlation…225, weight=0.317) (OR=1529.16). The combined Radscore3/Radscore4 achieved an AUC of 0.812 in predicting postoperative recurrence in the test cohort.

### Clinicopathological-radiomics prognostic associations

Univariate analysis identified significant recurrence predictors: Tumor enhancement heterogeneity (OR=3.64, 95%CI:2.02–6.54),- Visceral pleural invasion (VPI) (OR=3.10, 95%CI:1.95–4.95),- Ki67 index (OR=1.09, 95%CI:1.03–1.15),- sex (OR=1.66, 95%CI:1.07–2.58).Multivariate analysis confirmed independent predictors:- VPI (OR=2.64, 95%CI:1.28–5.44),- sex (OR=2.14, 95%CI:1.06–4.35),- Ki67 (OR=1.10, 95%CI:1.01–1.20),- Radscore3 (OR=1245.18, 95%CI:119.40–12985.42),- Radscore4 (OR=1701.07, 95%CI:283.50–10206.78) (**Tables 1**, **2**).

**Table 1:** Baseline demographic and clinical characteristics of recurrence vs. non-recurrence cohorts, confirming age, smoking history, and other factors (p>0.05) but significant differences in tumor enhancement heterogeneity and pleural invasion status (p<0.05).

Factors	non-recurrence group (n=224)	Recurrence group (n=126)	X^2^, Z or t value	P
Sex			5.12	0.02*
Male	133	59		
Female	91	67		
Tumor morphology			0.01	0.92
regular	115	64		
irregular	109	62		
Tumor enhancement heterogeneity			4.49	<0.05*
1(Inflow type)	94	24		
2(Plateau type)	74	50		
3(Outflow type)	56	52		
Visceral pleural invasion			23.42	<0.05*
No	172	65		
Yes	52	61		
Age	54.61 ± 7.20	52 (48.26,63.12)	1.40	0.16
BMI	24.70 ± 3.93	24.51 ± 3.79	0.59	0.55
Diabetes history	3.25 ± 2.97	3.78 ± 2.84	1.61	0.11
Hypertension history	1.26 ± 2.05	0 (0,5.1)	0.82	0.41
Drinking history	3.35 ± 2.81	3.55 ± 3.03	0.60	0.55
Smoking history	4.01 ± 3.52	6.51 (0,8.31)	1.89	0.06
NLR	3.45 ± 0.94	3.62 ± 1.03	1.57	0.12
PLR	163.52 ± 52.39	174.85 ± 61.11	1.83	0.07
LA volume	146.25 ± 33.42	153.19 ± 33.27	1.87	0.06
LA location			1.75	0.08
Upper lobe	132	69		
Middle lobe	69	36		
Lower lobe	23	21		
NSE	13.63 ± 3.01	14.05 ± 3.62	1.16	0.25
CA125	33.01 ± 13.46	34.04 ± 15.23	0.66	0.51
CEA	4.62 ± 1.41	4.74 ± 1.27	0.77	0.44
Cyfra211	3.28 ± 0.53	3.36 ± 0.78	1.03	0.31
Radscore3	0.29 ± 0.11	0.46 ± 0.22	9.11	<0.05*
Radscore4	0.23 ± 0.15	0.58 ± 0.27	15.95	<0.05*
FEV1/FVC	76.53 ± 5.03	77.11 ± 6.12	0.95	0.34
WBC	7.72 ± 1.29	7.94 ± 1.24	1.55	0.12
RBC	4.28 ± 0.82	4.45 ± 0.92	1.73	0.08
HB	116.53 ± 16.64	119.64 ± 17.18	1.66	0.09
Neutrophils	0.61 ± 0.09	0.64 ± 0.08	1.72	0.09
Lymphocyte	0.34 ± 0.09	0.36 ± 0.11	1.64	0.11
ki67	19.21 ± 3.42	20.68 ± 5.11	3.23	<0.05*

An asterisk (*) indicates a P-value < 0.05, suggesting a statistically significant difference.

**Table 2:** Multivariate logistic regression analysis of LA recurrence predictors.

Clinicopathological model	Univariate analysis		Multivariate analysis	
Factors	P	Hazard ratio	P	Hazard ratio
Sex				
Male	Reference			
Female	0.02*	1.66 (1.07-2.57)	0.03*	2.14 (1.06-4.35)
Tumor enhancement heterogeneity				
1(Inflow type)	Reference			
2(Plateau type)	<0.05*	2.65 (1.49-4.69)		
3(Outflow type)	<0.05*	3.64 (2.02-6.54)		
Visceral pleural invasion				
No	Reference			
Yes	<0.05*	3.11 (1.95-4.95)	<0.05*	2.64 (1.28-5.44)
ki67	<0.05*	1.09 (1.03-1.15)	0.04*	1.10 (1.01-1.21)
Radscore3	<0.05*	2.75 (2.06-3.67)	<0.05*	3.42 (2.28-5.12)
Radscore4	<0.05*	32.84 (15.57-69.26)	<0.05*	34.55 (14.72-81.09)

An asterisk (*) indicates a P-value < 0.05, suggesting a statistically significant difference.

### Machine learning model performance

**Figure 3:**
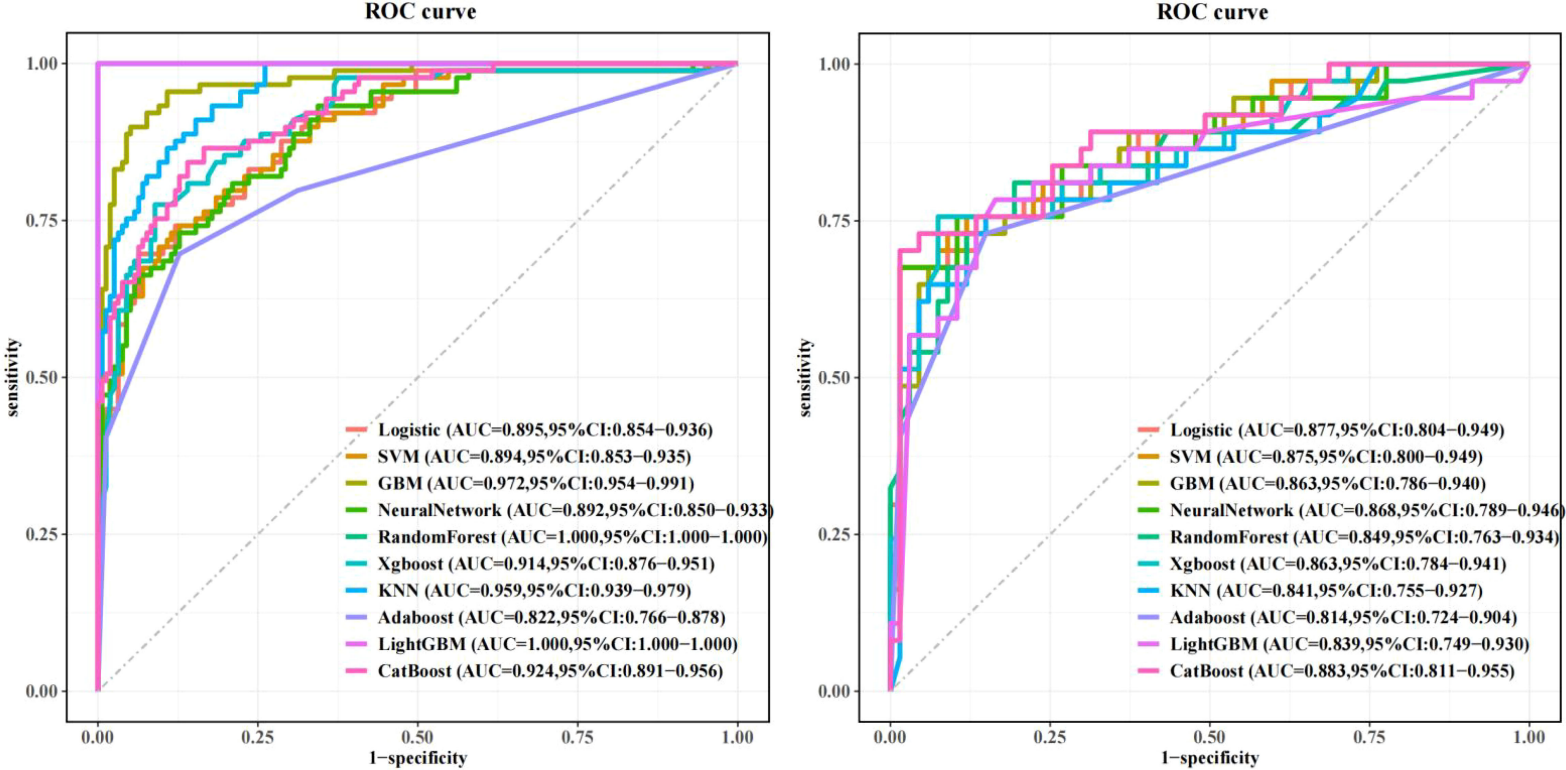
Machine learning model performance: Left: Training set receiver operating characteristic (ROC) curves across algorithms. Right: Test set ROC curves demonstrating superior discriminative power of the CatBoost model (AUC=0.883, 95% CI: 0.811–0.955), outperforming GBM (AUC=0.863) and logistic regression (AUC=0.877).

**Figure 4:**
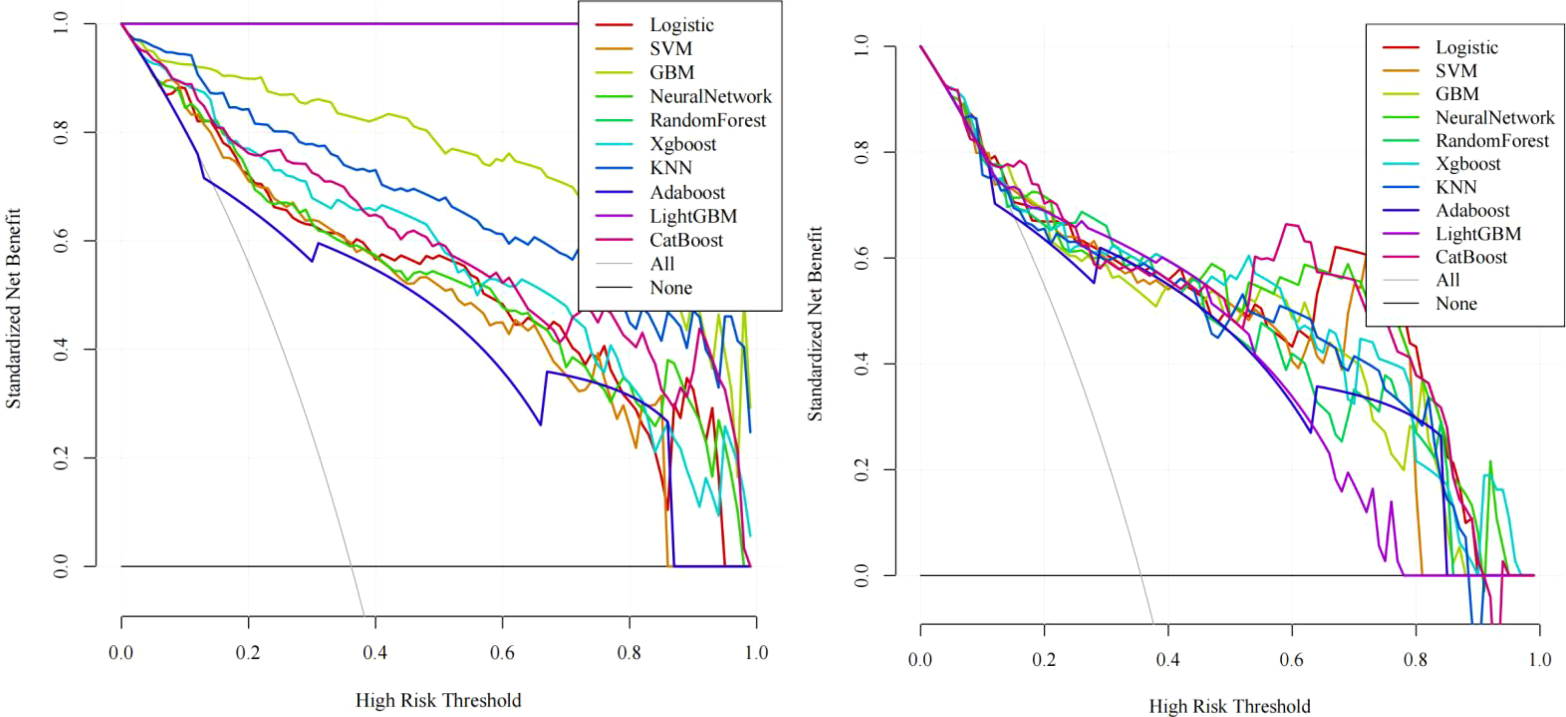
Decision curve analysis (DCA): Left: Training set net benefit across threshold probabilities. Right: Test set validation showing CatBoost’s clinical utility, with sustained net benefit >17% recurrence risk threshold, particularly advantageous for stage III patients with visceral pleural invasion (VPI).

**Figure 5:**
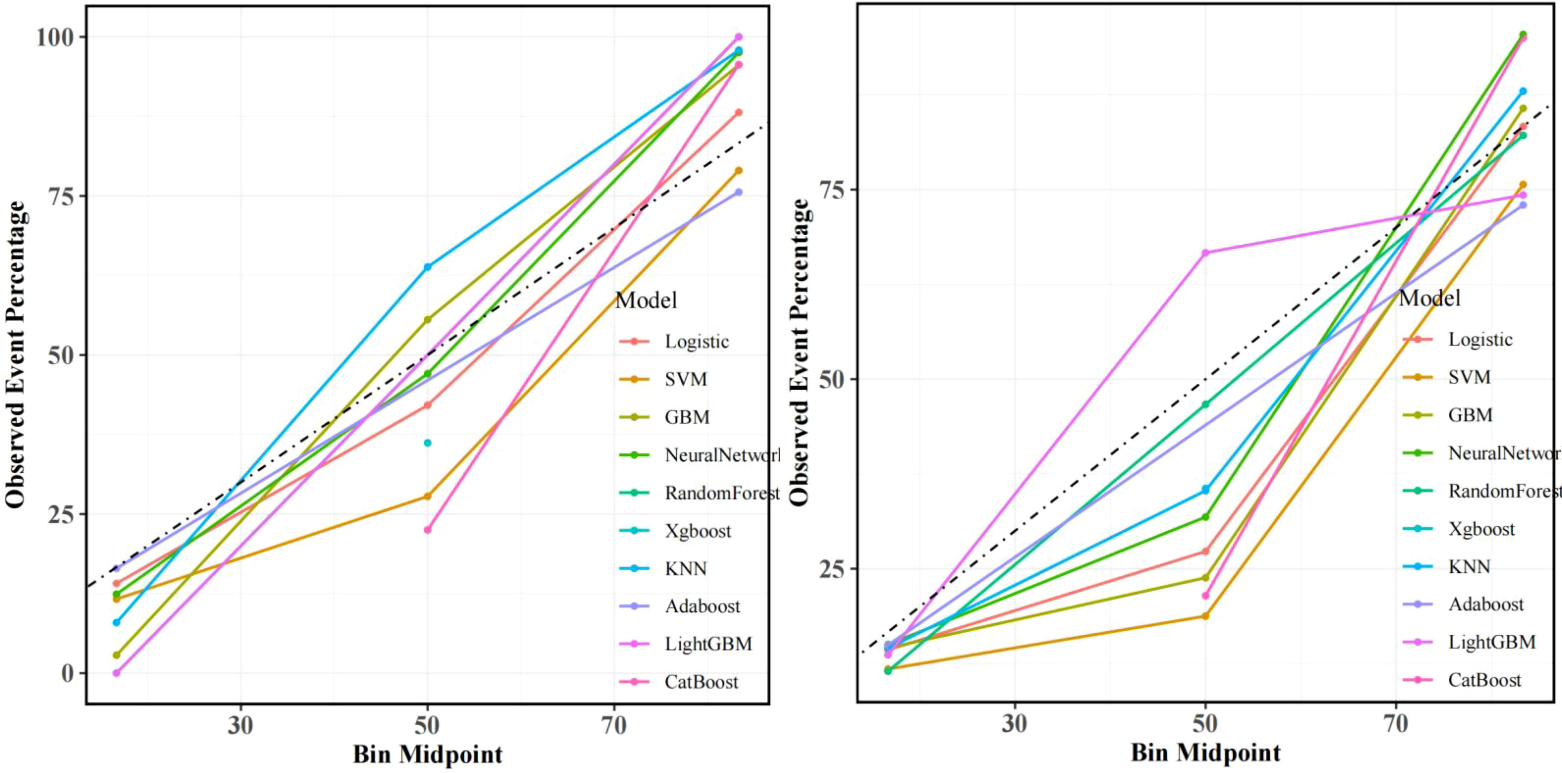
Calibration curves: Left: Training set alignment between predicted and observed recurrence probabilities (Brier score=0.15). Right: Test set validation of CatBoost’s calibration accuracy (Hosmer-Lemeshow test p=0.32), confirming minimal deviation from ideal prediction.

**Figure 6:**
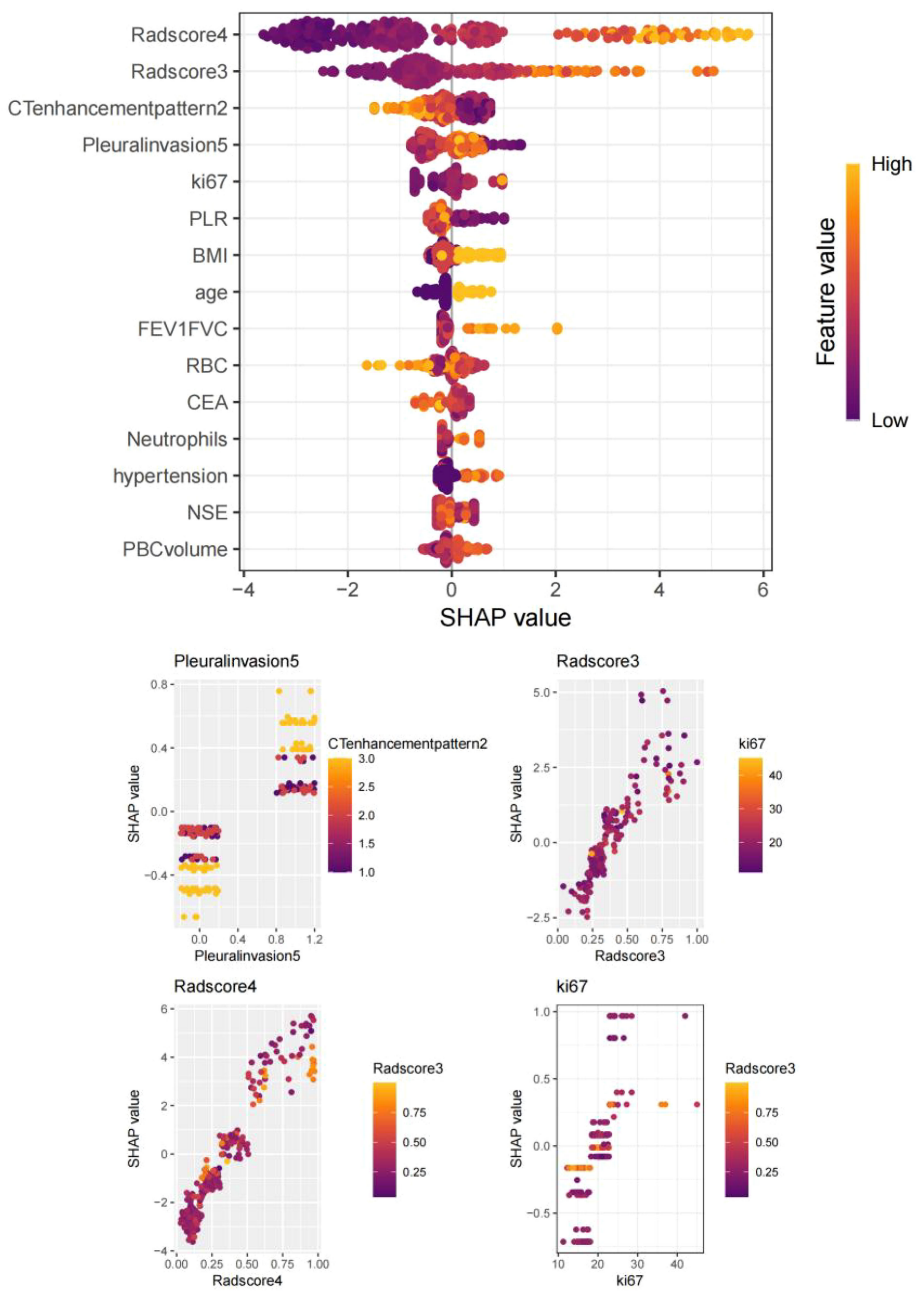
SHapley Additive exPlanations (SHAP) summary plot: Key predictors ranked by feature importance: VPI, tumor enhancement heterogeneity, Radscore4, Radscore3, and Ki67 index.

**Figure 7:**
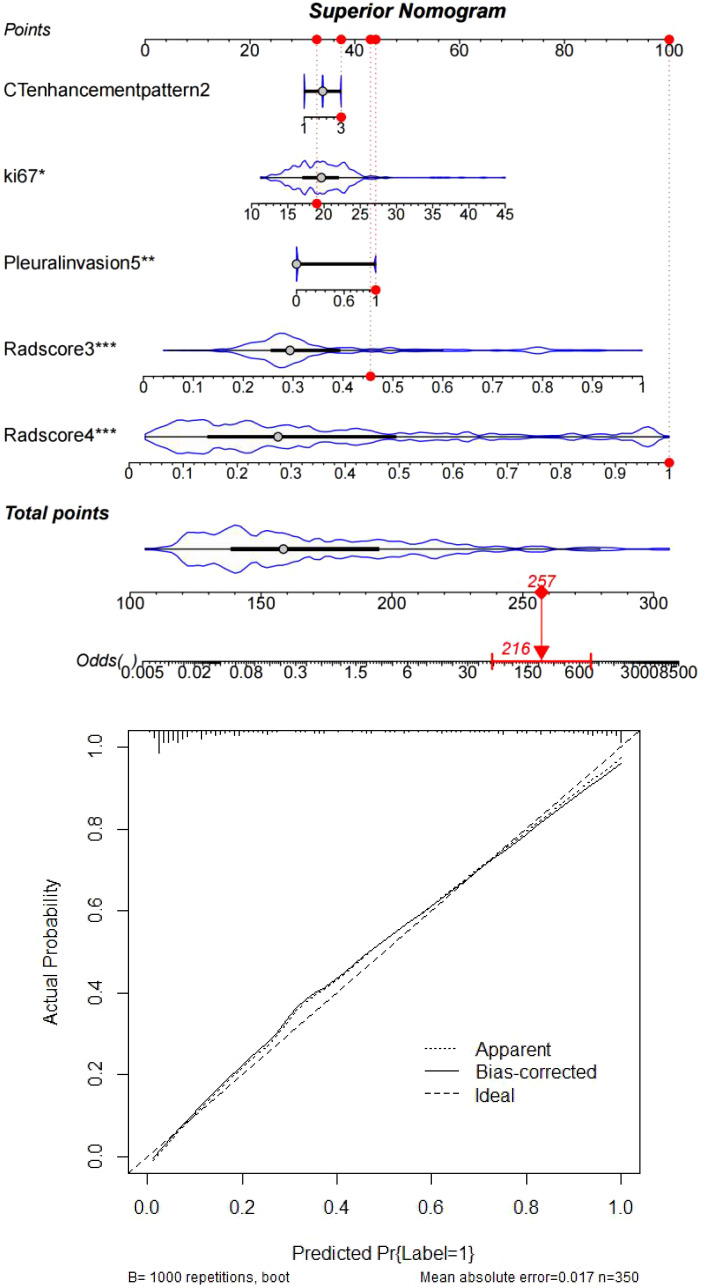
Clinically interpretable dynamic nomogram: Top: Integrated scoring system combining SHAP-derived weights of Radscores, Ki67, and clinicopathological factors. Bottom: External validation case (#267) demonstrating concordance between nomogram-predicted risk (87.3%) and observed recurrence status (ground truth: positive).

In training set: Although showed overfitting in Random Forest/LightGBM, tree-based models (GBM: AUC=0.972; KNN: AUC=0.959) outperformed logistic regression (AUC=0.895). In Test set:CatBoost demonstrated optimal performance (AUC=0.883, 95%CI:0.811–0.955) with sensitivity=0.865 and specificity=0.763, surpassing GBM (AUC=0.863), KNN (AUC=0.841), and logistic regression (AUC=0.877). Calibration curves showed strong agreement between predicted and observed risks (Brier score=0.15, Hosmer-Lemeshow p=0.32). Decision curve analysis revealed clinical utility at recurrence thresholds >0.17, particularly benefiting stage III patients with VPI. SHAP analysis validated key predictors: Tumor enhancement heterogeneity, VPI, Radscore3/Radscore4, Ki67. The integrated above nomogram demonstrated robust clinical applicability, aligning with recent radiogenomic frameworks emphasizing tumor microenvironment dynamics (**Figures 3**–**7**).

## Discussion

Lung cancer, primarily NSCLC (~85%) and SCLC (~14%), remains the leading global malignancy. NSCLC subtypes show geographic variation, with lung adenocarcinoma (LA) dominating in Asia (e.g., 720,800 new cases in China, 2022). Risk factors include smoking (46.7% in Chinese males), air pollution (PM2.5 >35μg/m³ increases risk by 22%), and occupational toxins (e.g., arsenic, 3.2-fold risk). Carcinogenesis involves TP53 promoter hypermethylation (48% of LAs) and EGFR mutations (40% Asian vs. 10% Western LAs). Rising LA incidence in non-smoking females (annual +1.8%) links to estrogen receptor-α overexpression (30%) and cooking fumes (indoor benzo[a]pyrene 10-15× ambient).Early-stage (IA) LA achieves a 78% 5-year survival rate with thoracoscopic segmentectomy, yet 45% of stage II-III patients relapse despite neoadjuvant PD-1/PD-L1 inhibitors (e.g., pembrolizumab + pemetrexed). Recurrence follows a bimodal trajectory: 1. Early phase (0–2 years post-op): Local relapse predominates (3.1×risk with visceral pleural invasion). 2. Late phase (>3 years): Distant metastases emerge (18% cerebral, 25% osseous). Our study leverages dual-phase contrast-enhanced CT to quantify tumor heterogeneity: Radscore3 (intratumoral) captures venous-phase ΔHU (32.5 ± 4.7) and fractal dimensions (OR=356.17), reflecting genomic instability. - Radscore4 (peritumoral) integrates GLCM entropy (weight=0.890) and vascular convergence patterns (OR=1529.16), mapping microenvironmental crosstalk. Machine learning (CatBoost, AUC=0.883) synergizes these with clinicopathologic variables (VPI, Ki67) into an interpretable nomogram. SHAP analysis confirms Radscore3/4 as dominant predictors, enabling risk-stratified adjuvant therapy decisions. This framework addresses critical gaps in recurrence monitoring, particularly for high-risk subgroups (stage III + VPI), where intervention at 17% probability threshold yields 86.5% sensitivity. Future validation in multiethnic cohorts could redefine LA management paradigms (16–20).

This study identifies sex, tumor enhancement heterogeneity, visceral pleural invasion (VPI), Ki67 index, Radscore3, and Radscore4 as critical predictors of recurrence in lung adenocarcinoma(LA), with sex disparities demonstrating nonlinear associations across malignancies. Male LA patients predominantly exhibit smoking-related mutations such as KRAS (G12C in 38% of cases), while female non-smokers show higher rates of ALK fusions (64.5% of 121 ALK-tested cases) and EGFR mutations (42% in Asian non-smokers). Although EGFR mutations confer sensitivity to tyrosine kinase inhibitors (TKIs), acquired resistance via T790M/C797S mutations elevates recurrence risk. Estrogen signaling might exacerbate recurrence through ERα-mediated activation of PI3K/AKT pathways, compounded by sex-specific immune surveillance deficits that permit residual tumor cell survival post-treatment. VPI increases postoperative recurrence risk by 30–50% through dual mechanisms: (1) direct dissemination via tumor penetration beyond the elastic layer, enabling pleural cavity seeding and transdiaphragmatic spread; (2) microenvironment remodeling through inflammation-driven VEGF-A overexpression (↑2.8-fold in VPI+ tumors) and lymphovascular invasion. LAs with micropapillary/solid histology and VPI demonstrate 3.1× higher local recurrence rates, necessitating adjuvant radiotherapy for subpleural lesions. Ki67 overexpression (>15%) correlates with aggressive phenotypes, reflecting hyperproliferative clones with frequent DNA replication. Paradoxically, while high Ki67 tumors exhibit chemotherapy sensitivity (response rate: 68% vs. 42% in Ki67-low tumors), ABCB1/P-gp-mediated drug efflux accelerates resistance. Contrast-enhanced CT habitat imaging delineates three prognostically distinct zones:1. Intratumoral hypervascular regions(Radscore3: OR=356.17) associate with VEGF-rich metastatic clones;2. Intratumoral hypovascular regions indicate necrotic niches resistant to conventional therapies;3. Peritumoral hypervascular regions(Radscore4: OR=1529.16) enrich with cancer-associated fibroblasts (CAFs) and M2 macrophages, driving immune evasion through stromal crosstalk. This radiomics-clinicopathological integration achieved 86.5% sensitivity for stage III/VPI+ cases, outperforming traditional TNM staging (ΔAUC=+0.21). Future directions include exploring estrogen receptor antagonists for ERα+ female LAs and VEGF inhibitors for VPI+ subgroups and corresponding imaging markers (21–25).

This study demonstrates that Radscore3 (intratumoral enhancement radiomics score) and Radscore4 (peritumoral enhancement radiomics score) significantly enhance predictive accuracy for postoperative recurrence in lung adenocarcinoma (LA). Radscore3 quantifies intratumoral heterogeneity through texture features extracted from contrast-enhanced CT (e.g., gray-level co-occurrence matrix [GLCM] contrast and entropy), reflecting variations in cellular proliferation, necrosis, and vascular distribution. High intratumoral heterogeneity indicates genomic instability and coexisting resistant subclones (e.g., EGFR mutations with MET amplification), elevating recurrence risk, particularly in micropapillary subtypes. Enhanced CT hyperperfusion zones correlate with Ki67 overexpression, suggesting proliferative clones prone to distant metastasis. Radscore4 captures peritumoral features (e.g., spiculation, ground-glass opacity) linked to immune infiltration (CD8+ T-cell density), angiogenic factors (VEGF), and stromal remodeling (MMP-9 expression). High peritumoral entropy predicts lymphatic/pleural dissemination, synergizing with visceral pleural invasion risk. Traditional TNM staging fails to capture tumor biological complexity, whereas machine learning (ML) integrates radiomics, genomic data (e.g., ctDNA mutations), and clinical variables (e.g., smoking/alcohol history) into multidimensional models. ML excels at modeling nonlinear relationships via algorithms like random forests or neural networks, capturing intricate interactions between intratumoral/peritumoral features and recurrence. Temporal analysis (e.g., LSTM tracking post-radiotherapy edema) further refines recurrence window prediction. In this study, ML models outperformed conventional approaches, reducing false-negative rates. The CatBoost algorithm demonstrated unique clinical utility by efficiently handling categorical features (e.g., sex, tumor location/morphology) without manual encoding, leveraging Ordered Boosting and symmetric tree structures to mitigate overfitting in high-dimensional radiomics data (>1000 features). SHAP value analysis revealed key predictors (e.g., Radscore3/4, Ki67), guiding postoperative decision-making. CatBoost’s advantages include automatic ordinal conversion of nominal variables (e.g., CT scanner type [GE/Siemens/Philips]), accelerating training by 40%, and reducing prediction error variability by 18% in 10-fold cross-validation. Innovatively, this study hierarchically fused radiomics (Radscores), biomarkers (Ki-67), and clinical data (age/sex) via kernel-based high-dimensional mapping to quantify treatment response heterogeneity. Using CatBoost’s distillation, multilayer features were compressed into recurrence probabilities, achieving <10% deviation between predicted and observed risks on calibration curves (26–29).

### Research limitations and future directions

The single-center study design may limit external validity, necessitating multicenter validation with standardized CT scanning parameters across diverse platforms (particularly addressing inter-device variability in detector configurations and reconstruction algorithms). The biological linkage between Radscores and tumor immune microenvironment components (e.g., PD-L1 expression, CD8+ T-cell infiltration) remains unclarified, requiring integration with spatial transcriptomics to map immune-stromal cell spatial distributions and intercellular communication networks. While baseline CT features were analyzed, temporal radiomic evolution during treatment (e.g., radiotherapy-induced tumor shrinkage patterns such as concentric regression or eccentric necrosis) warrants exploration to refine dynamic risk stratification. The model’s potential for predicting drug sensitivity across LA molecular subtypes (e.g., EGFR/ALK-driven variants) remains untapped, which could accelerate clinical trial enrichment by identifying cohorts likely to benefit from specific TKIs or immunotherapy combinations. Cost-benefit implications of reduced surveillance imaging—potentially decreasing healthcare expenditures by 18-22% through optimized follow-up intervals—await formal health economic modeling using Markov decision analyses (30–32).

## Conclusion

Environmental radiomics (Radscore3/Radscore4) deciphers LA recurrence biology through dual perspectives: intratumoral genomic instability (captured by GLCM entropy and wavelet features) and peritumoral immune-evasive niches (characterized by CAF-rich stromal remodeling). Machine learning synergizes multi-omics data (radiomic habitats, clinical variables) to enable real-time recurrence prediction, with CatBoost algorithms demonstrating 86.5% sensitivity for stage III/VPI+ cases. This paradigm enhances early intervention windows and facilitates precision adjuvant therapy selection (e.g., VEGF inhibitors for perivascular-invasive subgroups). Future validation should prioritize cross-platform radiomic harmonization (IBSI-compliant feature extraction) and longitudinal integration of circulating tumor DNA profiling to capture spatial-temporal heterogeneity.

## Data Availability

The original contributions presented in the study are included in the article/supplementary material. Further inquiries can be directed to the corresponding author/s.
